# Periocular necrotizing fasciitis following surgical site infection in aesthetic rhinoplasty: a report of two cases

**DOI:** 10.1186/s12348-026-00576-z

**Published:** 2026-03-27

**Authors:** Amirhossein Aghajani, Mohammad Taher Rajabi, Amin Zand, Zohreh Nozarian, Alireza Beikmarzehei, Elahe Havashki, Seyed Mohsen Rafizadeh

**Affiliations:** 1 Department of Oculo-Facial Plastic and Reconstructive Surgery, Farabi Eye Hospital, Tehran University of Medical Sciences, Qazvin Square, Tehran, 1336616351 Iran; 2 Department of Pathology, Farabi Eye Hospital, Tehran University of Medical Sciences, Tehran, Iran; 3 School of Medicine, Tehran University of Medical Sciences, Tehran, Iran

**Keywords:** Necrotizing fasciitis, Periocular, Periorbital, Facial, Rhinoplasty

## Abstract

**Purpose:**

To report a rare presentation of periocular necrotizing fasciitis (NF) following a surgical site infection after aesthetic rhinoplasty.

**Case presentation:**

We present two otherwise healthy patients who developed periocular swelling, erythema, and pain extending to the temporal region and nasal bridge, accompanied by areas of periocular necrosis. Neither patient had a history of trauma. One patient underwent an uneventful aesthetic rhinoplasty more than one month earlier, followed by a recent postoperative nasal hump shaving performed in an outpatient setting at the surgeon’s office. The second patient had a similar history of uneventful aesthetic rhinoplasty two weeks earlier. The laboratory risk indicator for necrotizing fasciitis (LRINEC) scores were 6 and 7, suggesting a high likelihood of the disease. Orbital computed tomography (CT) revealed severe preseptal soft tissue swelling extending to adjacent areas with no evidence of intraorbital involvement. A clinical diagnosis of periocular NF was established, and both patients were treated with intravenous broad-spectrum antibiotics, followed by prompt surgical debridement of necrotic tissue. Bacterial cultures revealed growth of *Streptococcus viridans* and *Staphylococcus* species. Both patients achieved complete recovery without recurrence or significant complications.

**Conclusions:**

Although periocular NF is rare, a high index of suspicion is essential for early diagnosis, particularly in patients with a recent history of facial surgery or trauma. Prompt recognition and management can prevent catastrophic outcomes, including orbital involvement and vision-threatening complications.

## Introduction

Necrotizing fasciitis (NF) is a rare, rapidly progressive, and potentially fatal infection of the superficial fascia and subcutaneous tissue, most commonly affecting the extremities [1]. Despite prompt medical and surgical intervention, the mortality rate associated with NF remains high, ranging from 10% to 40% [2, 3]. The most common predisposing factors include skin and soft-tissue infections, with *Group A Streptococcus* being the predominant pathogen [4]. The disease progresses more rapidly in immunocompromised individuals, such as patients with diabetes mellitus or those undergoing chemotherapy for malignant tumors [5, 6]. The pathogenesis of NF involves bacterial invasion of the subcutaneous tissues, rapid horizontal spread along deep fascial planes, and the release of bacterial toxins, leading to tissue ischemia and liquefactive necrosis [7]. Early diagnosis is challenging due to the absence of specific symptoms, and without timely intervention, the condition can rapidly progress to septic shock and death. Therefore, upon suspicion of NF, immediate management with broad-spectrum intravenous antibiotics and early, staged surgical debridement of necrotic tissue is critical [8, 9].

In rare cases, NF can affect the facial muscles and fascia, including the periocular and orbital regions [10]. When this occurs, the disease may progress rapidly, leading to severe complications such as cosmetic deformity, vision loss, or death [11, 12]. Previous literature suggests that the most common causes of periocular NF are trauma and odontogenic infections [10]. Facial surgical procedures are considered an uncommon predisposing factor for periocular NF [10, 13–16]. In this report, we present two cases of surgical site infections that progressed to periocular NF following recent aesthetic rhinoplasty.

## Case report

This study adheres to the CARE reporting guidelines [17]. All procedures were conducted in accordance with the principles outlined in the Declaration of Helsinki. Written informed consent for publication of the report and related images was obtained from the patients, and all patient details were fully de-identified. Ethical approval for case reports was not required by the institutional review board and Ethics Committee of Tehran University of Medical Sciences.

### Case 1

A 39-year-old otherwise healthy woman presented to the emergency department of Farabi Eye Hospital, Tehran, Iran, with progressive left-sided periorbital swelling, erythema, and pain that had begun one day prior to presentation. Her past medical history was unremarkable, and she denied any history of trauma. She had undergone uneventful aesthetic rhinoplasty at another medical center 45 days earlier. Additionally, she had recently undergone postoperative nasal hump shaving in an outpatient setting at her surgeon’s office three days before symptom onset.

On examination, her vital signs were as follows: blood pressure, 110/70 mmHg; axillary temperature, 38.2 °C; respiratory rate, 18 breaths per minute; and heart rate, 95 beats per minute. There were no signs of sinusitis or facial skin trauma, including periocular injury.

**Fig. 1:**
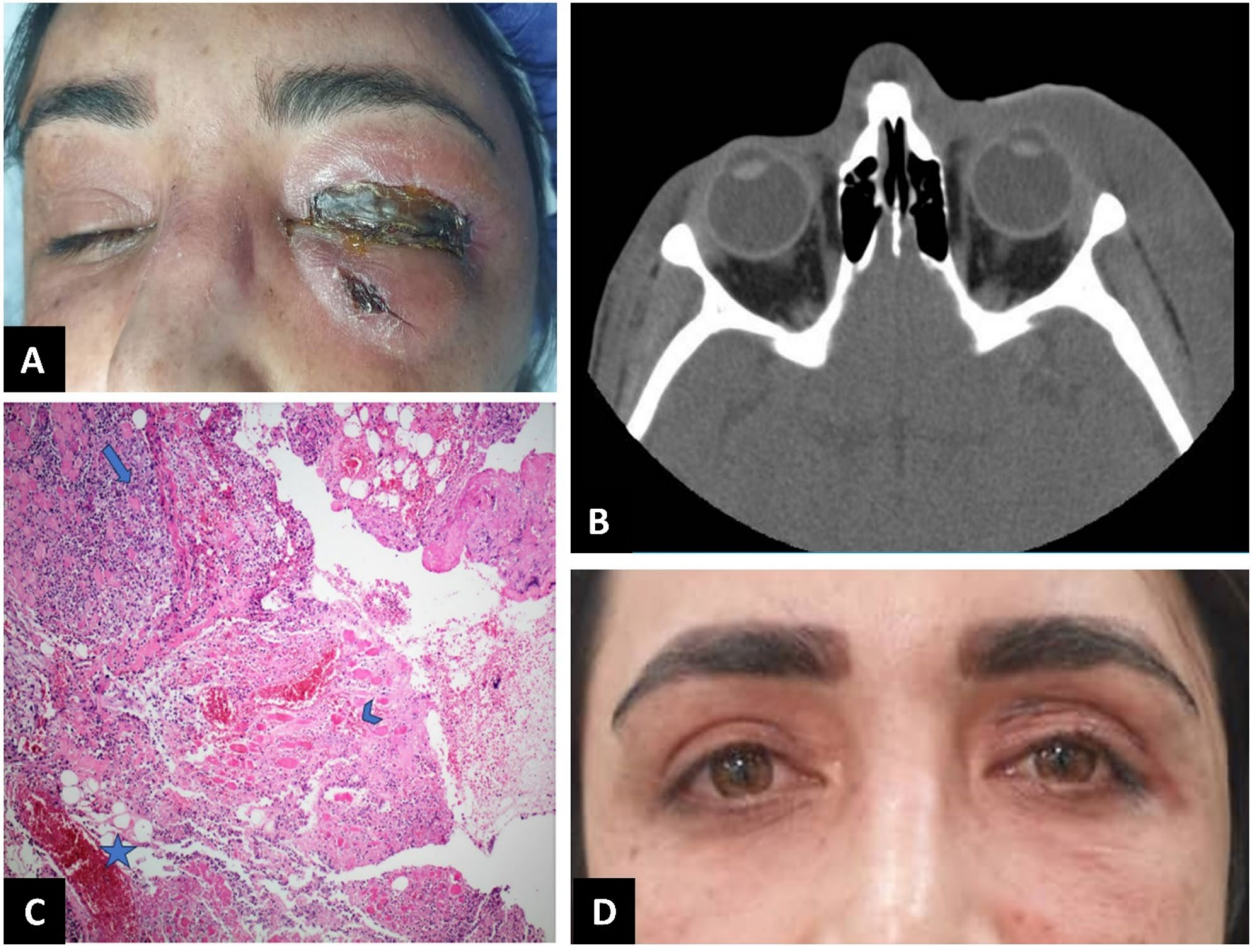
(**A**) Left periocular swelling extending to the temple area and nasal bridge, with necrotic tissue in the skin. Necrosis also involved the pretarsal and preseptal portions of the orbicularis oculi muscle in the upper eyelid and, to a lesser extent, the skin and orbital portion of the muscle in the lower eyelid. (**B**) Axial non-contrast orbital computed tomography scan showing severe left preseptal soft tissue swelling extending toward the temple area and nasal bridge, without evidence of bone involvement or intraorbital extension. (**C**) Histopathological examination revealing hemorrhage, adipose tissue containing gas inclusions (*star*), acute inflammatory infiltration (*arrow*), and necrotic striated muscle (*arrowhead*) (hematoxylin & eosin staining, ×100 magnification). (**D**) At the 8-month follow-up, complete resolution of the disease was observed, with no significant complications

On ophthalmic examination, her best-corrected visual acuity was 20/25 in the affected eye and 20/20 in the fellow eye. Pupillary reactions were normal, with no relative afferent pupillary defect detected. Periocular sensation on the affected side was diminished, and areas of induration and subcutaneous crepitus were palpable beneath the affected skin. The left periocular swelling extended to the temporal region and nasal bridge, with overlying skin necrosis. The necrosis affected the pretarsal and preseptal portions of the orbicularis oculi muscle in the upper eyelid and, to a lesser extent, the skin and orbital portion of the muscle in the lower eyelid (Fig. 1A). Eye movements were restricted to − 1 limitation in upgaze and adduction. There was no proptosis, and the orbit was neither tense nor resistant to retropulsion. Slit-lamp examination revealed mild conjunctival chemosis in the affected eye. Dilated fundus examination showed a clear vitreous with a normal retina and optic disc. The examination of the fellow eye was unremarkable.

Laboratory investigations revealed a white blood cell (WBC) count of 15.8 × 10³/mcL, with neutrophil predominance (58%), and a qualitative C-reactive protein (CRP) level of 2+. Her fasting blood glucose was 106 mg/dL, serum sodium was 133 mmol/L, hemoglobin was 10.6 g/dL, and creatinine was 0.6 mg/dL. Based on these results, her Laboratory Risk Indicator for Necrotizing Fasciitis (LRINEC) score was calculated to be 6 [18]. Blood cultures were negative for microbial growth. An orbital computed tomography (CT) scan without contrast revealed severe left preseptal soft tissue swelling extending to the temple area and nasal bridge, with no evidence of bony involvement or intraorbital extension. The paranasal sinuses were clear (Fig. 1B).

A diagnosis of necrotizing fasciitis was made based on the combination of clinical findings, imaging results, and laboratory data. Intravenous broad-spectrum antibiotic therapy, including meropenem (1 g three times per day), vancomycin (1 g twice per day), and clindamycin (600 mg three times per day), was initiated following consultation with the infectious disease team. The patient was taken emergently to the operating room for prompt surgical debridement of necrotic tissue. Intraoperatively, necrotic tissue in the left upper and lower eyelids was excised. The necrosis involved the anterior lamella (skin and portions of the orbicularis oculi muscle) without extension to the orbital septum or tarsal plate. The wounds were explored, and mucopurulent material was drained. After complete excision of the avascular necrotic tissue, the viable hemorrhagic soft tissues were irrigated with normal saline. Planned re-exploration and limited debridement were performed 24 and 48 h later. Pathological analysis of the discharge revealed gram-positive cocci, and bacterial cultures after 48 h showed growth of *Streptococcus viridans*. Further pathological evaluation indicated acute inflammatory reactions involving necrotic striated muscle and adipose tissue with gas formation (Fig. 1C).

Intravenous antibiotics were continued, along with warm compresses and massage. Oral prednisolone (25 mg/day) was added to the treatment regimen on hospital day 7. Gradual clinical improvement in periorbital and temple area swelling was achieved over 21 days of intravenous antibiotic treatment. At this time, repeat laboratory investigations were within normal limits, and the patient was discharged on oral clindamycin (300 mg three times per day) and amoxicillin/clavulanic acid (625 mg three times per day). At two months, the patient underwent reconstruction of the anterior lamella defect in the left upper eyelid using a skin graft from the contralateral upper eyelid. No recurrence of symptoms occurred in subsequent follow-ups. An eight-month follow-up examination revealed near-complete resolution of the initial signs, with no further complications (Fig. 1D).

### Case 2

A 20-year-old otherwise healthy woman presented to the emergency department of Farabi Eye Hospital, Tehran, Iran, with a four-day history of progressive right periorbital swelling, erythema, and pain. She had undergone uneventful aesthetic rhinoplasty at another medical center two weeks earlier. Her medical history was unremarkable, and she denied any history of trauma.

On examination, her vital signs were as follows: blood pressure, 120/85 mmHg; axillary temperature, 38.8 °C; respiratory rate, 20 breaths per minute; and heart rate, 110 beats per minute. There were no signs of sinusitis or facial skin trauma, including periocular injury.

**Fig. 2:**
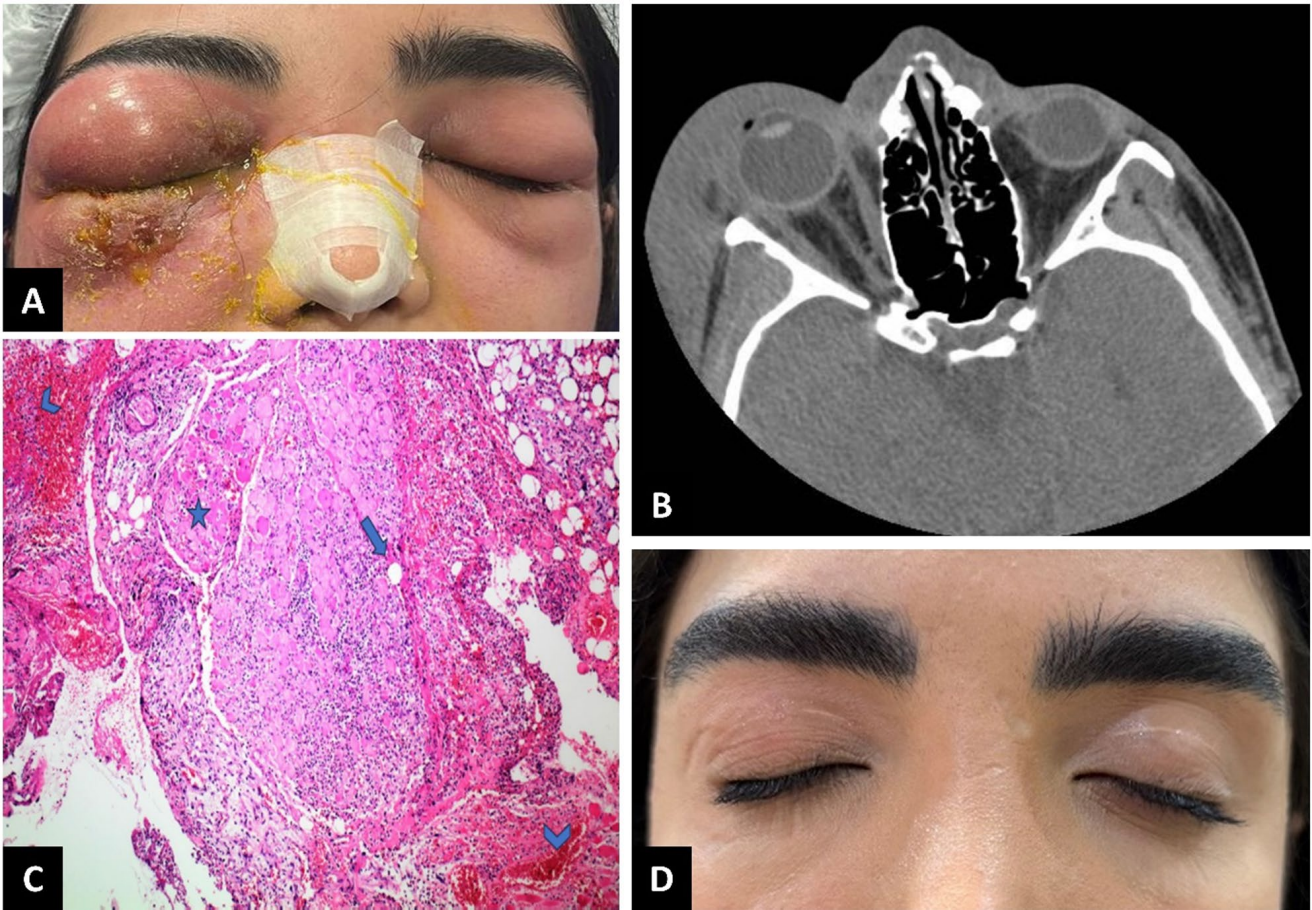
(**A**) Right periocular swelling extending to the temple area, nasal bridge, cheek, and contralateral periocular regions, with necrotic tissue involving the skin and multiple portions of the orbicularis oculi muscle in the lower eyelid. (**B**) Axial non-contrast computed tomography scan demonstrating severe soft tissue thickening in the periorbital and temporal regions, extending to the nasal bridge and contralateral periocular area. No signs of bone involvement or intraorbital extension were observed. (**C**) Histopathological analysis showing gas inclusions (*arrow*) between necrotic and inflamed muscle fibers (*star*) within infected and hemorrhagic tissue (*arrowhead*) (hematoxylin & eosin staining, ×100 magnification). (**D**) At the 2-year follow-up, the patient exhibited complete resolution without significant complications

On ophthalmic examination, her best-corrected visual acuity was at least 20/50 in the affected eye and 20/25 in the fellow eye. However, due to severe eyelid edema, visual acuity in the affected eye may have been underestimated. Pupillary reactions were normal, with no relative afferent pupillary defect detected. Periocular sensation on the affected side was diminished, and areas of induration and subcutaneous crepitus were palpable beneath the affected skin. The right periocular swelling extended to the temporal region, nasal bridge, cheek, and contralateral periocular area, with overlying skin necrosis, as well as necrosis involving multiple portions of the orbicularis oculi muscle in the lower eyelid (Fig. 2A). To the extent permitted by patient cooperation, eye movement was restricted to − 2 in upgaze and − 1 in the remaining cardinal positions. The orbit was not tense on palpation. Slit-lamp examination revealed mild conjunctival chemosis in the affected eye. Due to severe eyelid swelling and limited patient cooperation, a complete dilated fundus examination was challenging but appeared grossly unremarkable. The examination of the fellow eye was unremarkable except for mild periocular swelling.

Laboratory investigations revealed an elevated WBC count of 27.3 × 10³/mcL, with neutrophil predominance (64%), and a qualitative CRP level of 2+. Fasting blood glucose was 120 mg/dL. Serum sodium was 132 mmol/L, hemoglobin was 10.3 g/dL, and creatinine was 0.8 mg/dL. Based on these results, her LRINEC score was calculated to be 7 [18]. A non-contrast orbital computed tomography (CT) scan revealed severe soft tissue thickening in the periorbital and temporal regions of the affected side, extending to the nasal bridge and contralateral periocular area. No evidence of bony involvement or intraorbital extension was identified, and the paranasal sinuses were clear (Fig. 2B).

Given the diagnosis of periocular NF, intravenous broad-spectrum antibiotics—including meropenem (1 g three times per day), vancomycin (1 g twice per day), and clindamycin (600 mg three times per day)—were administered following consultation with the infectious disease team. Urgent surgical debridement of the infected necrotic tissue was performed. Pathological analysis of the discharge revealed gram-positive cocci, and bacterial cultures after 48 h showed growth of *Staphylococcus*. Further pathological evaluation indicated an extensive suppurative inflammatory process with striated muscle necrosis and areas of gas formation (Fig. 2C).

Intravenous antibiotics were continued, along with warm compresses and massage. Oral prednisolone (25 mg/day) was introduced into the treatment regimen on hospital day 10. Progressive clinical improvement was observed over 21 days of intravenous antibiotic therapy. The patient was subsequently discharged with oral clindamycin (300 mg three times per day) and amoxicillin/clavulanic acid (625 mg three times per day). No recurrence of symptoms was noted in subsequent follow-ups. At the two-year follow-up, the patient exhibited complete resolution of the condition without any significant complications (Fig. 2D).

## Discussion

NF is a severe infectious disease with a rapid course and a reported mortality rate of up to approximately 35% [13, 19–21]. Its progressive inflammatory and necrotizing nature can affect soft tissues, including muscle, fascia, and peripheral nerves, and may extend to adjacent structures from the primary site of infection [7]. Although facial and periocular involvement is rare—possibly due to the rich blood supply in these areas—the consequences can be catastrophic, particularly if the infection extends into the orbital cavity. In such cases, complications may include vision loss, globe necrosis, or even the need for aggressive surgical debridement, including orbital exenteration [22–25].

The most common pathogens responsible for NF include *Group A beta-hemolytic streptococci*,* methicillin-resistant Staphylococcus aureus (MRSA)*,* Klebsiella pneumoniae*,* Pseudomonas aeruginosa*,* Staphylococcus epidermidis*,* Streptococcus milleri group*,* Acinetobacter sp.*,* Enterobacter cloacae*, and fungi [10]. Consistent with previous reports, *Streptococcus viridans* was identified in Case 1, while *Staphylococcus* species were responsible for the infection in Case 2.

Pertea et al. analyzed cases of periorbital facial NF, and identified the most common predisposing factors as trauma (41%), odontogenic infections (27%), dermatological conditions (14%), and surgical procedures (10%), with an additional 10% attributed to other causes. Notably, in 38% of cases, no identifiable source was found [10]. In both cases, the patients had a history of recent aesthetic rhinoplasty before the onset of symptoms. While rhinoplasty is a complex and technically challenging procedure, complications are rare, with infections occurring in only 0.1%–0.5% of cases [26]. These infections typically manifest at the surgical site, and periocular infections secondary to rhinoplasty have been reported only rarely, most often as necrotizing periorbital cellulitis [15, 16]. Moscona et al. described a case of severe eyelid swelling and superficial necrosis one day after septorhinoplasty [15]. Similarly, Haik et al. reported a case of necrotizing periorbital cellulitis occurring two days post-rhinoplasty, presenting with eyelid swelling and superficial necrosis in the lower eyelid region [16]. Both cases were caused by *beta-hemolytic streptococci* and responded well to systemic antibiotics and conservative local treatment, without requiring surgical debridement due to their mild severity [15, 16]. Other reported cases of post-facial surgical NF have typically manifested shortly after surgery [13, 14]. In contrast, our cases demonstrated delayed-onset surgical site infections progressing to periocular NF. Case 1 was infected with *Streptococcus viridans* and Case 2 with *Staphylococcus* species, both of which progressed to clinically overt and severe NF—an uncommon scenario. In Case 1, we hypothesized that the probable source of delayed infection was postoperative nasal hump shaving performed in the surgeon’s office. Although NF is more common in immunocompromised patients with comorbidities such as diabetes mellitus, malignancies, or immunosuppression [5, 6], both of our patients were otherwise healthy. Similarly, Pertea et al. reported that only 50% of patients with periorbital facial NF had an identifiable predisposing comorbidity [10].

Facial NF most commonly affects the periorbital region, as seen in our cases [10]. The most frequently reported symptoms include soft tissue swelling, pain, fever, edema, erythema, and purulent discharge [10]. Our patients initially presented with periocular swelling and pain that extended to adjacent facial structures, with areas of soft tissue necrosis in the periocular region. Diagnosing periocular NF in its early stages is challenging, as initial signs may closely resemble severe preseptal cellulitis. However, certain features—including skin necrosis, bullae, crepitation, severe tenderness, and hypoesthesia—should raise suspicion for NF [27]. The LRINEC scoring system, which evaluates CRP levels, WBC count, serum sodium, hemoglobin, blood glucose, and creatinine levels, can aid in diagnosing and assessing disease severity [18]. A score of ≥ 6 is considered suggestive of NF, while a score ≥ 9 is associated with a poor prognosis [28]. In cases of periorbital facial NF, Pertea et al. reported LRINEC scores ranging from 6 to 10 [10]. Similarly, our patients had scores of 6 (Case 1) and 7 (Case 2). Imaging modalities can help differentiate early-stage NF from other conditions, such as cellulitis. Ultrasonography may reveal localized fluid or gas collection in the deep fascia [29]. CT and magnetic resonance imaging can detect soft tissue thickening, fluid collections, and gas bubbles—features highly suggestive of NF [30, 31].

The recommended antibiotic regimen for NF typically includes a combination of broad-spectrum antibiotics such as imipenem, meropenem, daptomycin, and piperacillin/tazobactam, often with the addition of clindamycin [32]. While mild cases may respond to medical therapy alone, antibiotic penetration into necrotic, avascular tissue is often inadequate. Thus, successful treatment usually requires a combination of antimicrobial therapy, fluid resuscitation, electrolyte management, and urgent surgical debridement, as performed in our cases [8, 9]. Based on clinical signs, frequent and aggressive debridement should be conducted, and tissue samples must be sent for microbiological analysis [33]. In cases with gas formation, hyperbaric oxygen therapy may be beneficial [34, 35]. Early debridement of necrotic tissue is critical for preventing thrombosis and extensive skin loss. The infected site should be left open and not covered until complete healing is achieved [36]. Pertea et al. reported that surgical debridement was required in all cases of facial NF, with many patients requiring skin grafting or flap reconstruction—consistent with our Case 1, which underwent a skin graft from the contralateral eyelid [10].

In conclusion, although periorbital NF is uncommon, clinicians should maintain a high index of suspicion in patients presenting with suggestive signs and symptoms. A detailed history of recent trauma or surgery can facilitate early diagnosis and timely intervention. Prompt treatment—including systemic broad-spectrum antibiotics and serial surgical debridement of necrotic tissue—is essential to prevent devastating complications such as orbital involvement, which may lead to vision loss, or even exenteration.

## Data Availability

No datasets were generated or analysed during the current study.
